# A systematic review and meta-analysis of minimally invasive total mesorectal excision versus transanal total mesorectal excision for mid and low rectal cancer

**DOI:** 10.3389/fonc.2023.1167200

**Published:** 2023-06-12

**Authors:** Du Yong Gang, Lin Dong, Zhang DeChun, Zhang Yichi, Lu Ya

**Affiliations:** ^1^ Department of Gastrointestinal Surgery, Pengzhou People's Hospital, Chengdu, Sichuan, China; ^2^ Department of Urology, Pengzhou People's Hospital, Chengdu, Sichuan, China; ^3^ Department of Respiratory Medicine, First Affiliated Hospital of Chengdu Medical College, Chengdu, Sichuan, China

**Keywords:** minimally invasive total mesorectal excision, transanal total mesorectal excision, mid and low-rectal cancer, systematic review, meta-analysis

## Abstract

**Background:**

Minimally invasive total mesorectal excision (MiTME) and transanal total mesorectal excision (TaTME) are popular trends in mid and low rectal cancer. However, there is currently no systematic comparison between MiTME and TaTME of mid and low-rectal cancer. Therefore, we systematically study the perioperative and pathological outcomes of MiTME and TaTME in mid and low rectal cancer.

**Methods:**

We have searched the Embase, Cochrane Library, PubMed, Medline, and Web of Science for articles on MiTME (robotic or laparoscopic total mesorectal excision) and TaTME (transanal total mesorectal excision). We calculated pooled standard mean difference (SMD), relative risk (RR), and 95% confidence intervals (CIs). The protocol for this review has been registered on PROSPERO (CRD42022374141).

**Results:**

There are 11010 patients including 39 articles. Compared with TaTME, patients who underwent MiTME had no statistical difference in operation time (SMD -0.14; CI -0.31 to 0.33; I^2=^84.7%, P=0.116), estimated blood loss (SMD 0.05; CI -0.05 to 0.14; I^2=^48%, P=0.338), postoperative hospital stay (RR 0.08; CI -0.07 to 0.22; I^2=^0%, P=0.308), over complications (RR 0.98; CI 0.88 to 1.08; I^2=^25.4%, P=0.644), intraoperative complications (RR 0.94; CI 0.69 to 1.29; I^2=^31.1%, P=0.712), postoperative complications (RR 0.98; CI 0.87 to 1.11; I^2=^16.1%, P=0.789), anastomotic stenosis (RR 0.85; CI 0.73 to 0.98; I^2=^7.4%, P=0.564), wound infection (RR 1.08; CI 0.65 to 1.81; I^2=^1.9%, P=0.755), circumferential resection margin (RR 1.10; CI 0.91 to 1.34; I^2=^0%, P=0.322), distal resection margin (RR 1.49; CI 0.73 to 3.05; I^2=^0%, P=0.272), major low anterior resection syndrome (RR 0.93; CI 0.79 to 1.10; I^2=^0%, P=0.386), lymph node yield (SMD 0.06; CI -0.04 to 0.17; I^2=^39.6%, P=0.249), 2-year DFS rate (RR 0.99; CI 0.88 to 1.11; I^2=^0%, P = 0.816), 2-year OS rate (RR 1.00; CI 0.90 to 1.11; I^2=^0%, P = 0.969), distant metastasis rate (RR 0.47; CI 0.17 to 1.29; I^2=^0%, P = 0.143), and local recurrence rate (RR 1.49; CI 0.75 to 2.97; I^2=^0%, P = 0.250). However, patients who underwent MiTME had fewer anastomotic leak rates (SMD -0.38; CI -0.59 to -0.17; I^2=^19.0%, P<0.0001).

**Conclusion:**

This study comprehensively and systematically evaluated the safety and efficacy of MiTME and TaTME in the treatment of mid to low-rectal cancer through meta-analysis. There is no difference between the two except for patients with MiTME who have a lower anastomotic leakage rate, which provides some evidence-based reference for clinical practice. Of course, in the future, more scientific and rigorous conclusions need to be drawn from multi-center RCT research.

**Systematic review registration:**

https://www.crd.york.ac.uk/PROSPERO, identifier CRD42022374141.

## 1 Introduction

Rectal cancer ranks third among the most common malignant tumors worldwide (1), and about 65% of rectal cancer is in the middle to low position. Total mesorectal excision (TME) is currently the standard surgical procedure for rectal cancer (2, 3). Some factors related to the recurrence, prolonged operation time (OP), and increased complications of rectal cancer have been identified, including male patients, pelvic stenosis, obese patients, and tumor size (4, 5). With the advancement of medical engineering technology, minimally invasive total mesorectal excision (MiTME) has gradually replaced open total mesorectal excision (OpTME) (6). Compared to OpTME, MiTME has a clear field of vision and a more precise operation process, which can obtain high-quality TME (7). However MiTME, especially in patients with difficult pelvic conditions, may not provide a clearer view and high-quality TME, and taTME has emerged, overcoming the drawbacks of previous MiTME techniques (8). There is currently a lack of meta-analysis that integrates laparoscopic and robotic versus transanal total mesorectal excision (TaTME). Therefore, the purpose of the meta-analysis is to analyze the perioperative, postoperative, and oncology outcomes of MiTME versus TaTME for mid and low rectal cancer.

## 2 Methods

### 2.1 Protocol and guidance

The study was performed according to Preferred Reporting Items for Systematic Reviews and the meta-analysis (PRISMA) (9) and the quality evaluation of this article was scored using the Newcastle-Ottawa Scale (NOS) score. The protocol for this review has been registered on PROSPERO (CRD42022374141).

### 2.2 Search strategy

This study involved literature published in the Embase, PubMed, Cochrane Library, Medline, and Web of Science up to September 18, 2022. We defined the eligibility criteria according to the population(P), intervention(I), comparator(C), outcome, and study design approach(O). P: The patients with mid and low rectal cancer. I: undergoing MiTME. C: TaTME was performed as a comparator. O: one or more of the following outcomes: perioperative period, postoperative indices, and oncologic outcomes. The search terms included (laparotomy OR laparoscopy OR laparoscopic OR minimally invasive OR robot OR robotic) AND (transanal OR perineal OR natural orifice) AND (colorectal cancer OR rectal cancer OR mesorectal excision OR TME OR proctectomy OR anterior resection OR abdominoperineal excision). The search strategy was not limited by language or year. The ethics or institutional review committee did not request it due to the study being designed as a systematic review and meta-analysis.

### 2.3 Inclusion and exclusion criteria

We have included the literature by the following criteria. Comparative data were available on the treatment of mid and low-rectal cancer through MiTME (RaTME and LaTME) and TaTME. Outcome indexes should include at least one of the following, perioperative period, postoperative indices, and oncologic outcomes. Any study which did not confirm the above inclusion criteria was excluded.

### 2.4 Data extraction and outcome measures

Two researchers (L.D. and Y.L.) independently reviewed the retrieved literature by the inclusion and exclusion criteria. The third researcher (Z.Y.C) was asked to participate in the discussion to decide whether to include when disagreements were encountered. The extracted data included the first author, publication, country, study type, group, age, follow-up, tumor height, and tumor size (if mentioned) (**Table 1**).

**Table 1:** The main characteristics of included studies.

Author	Publication	Country	Study period	Study design	Group	Cases	Age	BMI(Body mass index) (kg/m2)	Tumor size	Tumor height	Follow-up (months)	Confounders adjustment	NOS score(max:9)	
Alhanafy et al., 2020 (10)	Diseases of the colon and rectum	South Korea	2014-2017	Retrospective	laTME	202	61.50±11.20	24.10±3.40			34.0 (0.7–63.3)	Yes (propensity score matching)	8	
					taTME	202	62.40±9.98	24.02±3.10			34.0 (0.7–63.3)			
Bedrikovetski et al., 2020 (11)	Dis Colon Rectum	Australia	2007-2018	Retrospective	RoTME	117	63 (31–87)			7 (0–15)		No	8	
					laTME	1269	66 (18–97)			8 (0–18)				
					taTME	85	64 (32–86)			7 (1–15)				
Bjoern et al., 2019 (12)	J Gastrointest Surg	Denmark	2010-2017	Prospective	laTME	36	62.42 ± 10.146	25.45 ± 4.811		8.14 ± 1.885	75.08	No	7	
					taTME	49	64.88 ± 9.645	26.57 ± 3.476		8.35 ± 1.727	22.69			
Bjoern et al., 2022 (13)	Int J Colorectal Dis	Denmark	2016-2019	Retrospective	la/RoTME	92	67.5 (43.7–89.4)	Normal 18.5–24.9: 32 (35.2)			13.5	No	8	
					taTME	115	69 (39–95)	Normal 18.5–24.9: 51 (44.3)			13.5			
Chang et al., 2018 (14)	Journal of laparoendoscopic & advanced surgical techniques	China	2014-2017	Prospective	laTME	23	62.9 – 12.6	25.0 – 3.9	3.3 – 1.6	5.9 – 1.1		Yes (propensity score matching)	8	
					taTME	23	62.4 – 12.9	25.8 – 4.3	3.2 – 2.1	4.3 – 1.4				
Chen et al., 2019 (15)	Asian journal of surgery	China	2008-2018	Retrospective	laTME	64	64.0 12.2	24.6 3.3	3.2 1.5		37.5 23.7	No	8	
					taTME	39	62.0 14.9	25.4 4.0	3.6 2.2		17.5 8.8			
Detering et al., 2019 (16)	Journal of the American College of Surgeons	Netherlands	2015-2017	Prospective	laTME	396	>75y,23.2					Yes (propensity score matching)	9	
					taTME	396	>75y,18.2							
Dou et al., 2019 (17)	Zhonghua Wei Chang Wai Ke Za Zhi	China	2016-2017	Retrospective	laTME	53	62.0(33.0-73.0)	22.2(16.7~27.7)			16.2(12.1~30.4)	No	6	
					taTME	54	57.5(26.0~77.0)	21.5(17.8~33.2)			17.9(12.1~30.4)			
Fernandez-Hevia et al., 2015 (18)	Annals of Surgery	Spain	2011-2013	Retrospective	laTME	37	69.5 ± 10.5		2.7 ± 1.5			Yes (propensity score matching)	9	
					taTME	37	64.5 ± 11.8		2.6 ± 1.4					
Grass et al., 2021 (19)	International journal of colorectal disease	Germany	2014-2018	Prospective	RoTME	55	59.2±11.9	27.2± 5.3			25.9 ± 13.1	No	8	
					taTME	65	66.6± 10.4	25.4± 4.0			25.7 ± 11.7			
Hol et al., 2021 (20)	The British journal of surgery	The Netherlands	2015-2017	Retrospective	RoTME	344	67(10.6)	26(4.0)				No	7	
					laTME	490	68(9.8)	26(4.4)						
					taTME	244	66(11.0)	26(4.2)						
Jang et al., 2021 (21)	Asian journal of surgery	Korea	2009-2019	Retrospective	laTME	182	66.68 (11.266)	23.12 (3.894)	5.0 (2.095)			No	8	
					taTME	38	68.87 (12.034)	22.82 (3.149)	3.73 (2.974)					
Law et al., 2019 (22)	Surg Endosc	China	2014-2017	Prospective	RoTME	40	69.5 (45–88)		35 (0–90)			Yes (propensity score matching)	7	
					taTME	40	64.5 (40–79)		25 (0–60)					
Lee et al., 2018 (23)	Ann Coloproctol	Korea	2013-2014	Prospective	RoTME	24	<60: 18	23.6 ± 3.00		5.2 ± 1.99	22	Yes (propensity score matching)	7	
					taTME	21	<60:10	24.4 ± 3.44		6.1 ± 1.63	20.1			
Lee et al., 2019 (24)	Annals of Surgery	Korea	2011-2017	Retrospective	RoTME	370	62.5 ±11.1	25.8 (4.0)	3.0 (2.1)	5.6 (2.6)		Yes (propensity score matching)	9	
					taTME	226	62.1±11.7	26.1 (3.8)	2.8 (1.9)	5.6 (2.5)				
Li et al., 2022 (25)	Surg Endosc	China	2014-2019	Retrospective	laTME	106	56 ± 12 (26–79)	22:9 ± 3:2 (16.9-34.3)	2:8 ± 2:0 (0-8.0)		30:29 ± 13:439 (1–73)	Yes (propensity score matching)	7	
					taTME	106	55 ± 12 (23–78)	23:0 ± 2:9 (17.2-32.3)	3:0 ± 1:3 (0.3-6.6)		21:80 ± 18:153 (1–121)			
Li et al., 2021 (26)	Tech Coloproctol	China	2014-2018	Prospective	laTME	30	p = 0.732	22.6 (19.3–27.6)			22.2	Yes (propensity score matching)	8	
					taTME	30		27.3 (24.4–32.5)			13.8			
Liu et al., 2022 (27)	Annals of Surgery	China	2016-2021	Prospective	laTME	545	60 (52–67)	22.8 (20.9-24.8)				No	9	
					taTME	544	58 (50–67)	22.9 (20.7-24.9)						
Mora et al., 2018 (28)	Cir Cir	Spain	2011-2014	Prospective	laTME	15	64					No	7	
					taTME	16	59.95							
Munini et al., 2021 (29)	Int J Colorectal Dis	Switzerland	2012-2019	Prospective	laTME	35	69.0 (59.0–74.0)	25.1 (24.0–30.8)	2.5 (2.0–3.9)		49.5 (22.6–68.5)	Yes (propensity score matching)	7	
					taTME	35	67.0 (60.1–73.6)	27.2 (23.8–28.9)	2.5 (1.5–3.5)		30.6 (20.2–39.8)			
Ong et al., 2021 (30)	Am J Surg	USA	2014-2019	Retrospective	laTME	30	57.9 ± 10.9	28.7 ± 5.5			20.4 ± 15.9	No	8	
					taTME	20	61.4 ± 11.3	28.3 ± 5.2			24.9 ± 12.7			
Ose et al., 2021 (6)	Colorectal Disease	Denmark	2014-2018	Prospective	RoTME	713	67.28 ± 10.074	26.15 ± 4.405				No	8	
					laTME	1163	67.61 ± 10.254	26.52 ± 7.199						
					taTME	312	65.65 ± 10.038	26.08 ± 4.419						
Ourô et al., 2022 (31)	Tech Coloproctol	Portugal	2016-2018	Retrospective	laTME	39	69 (61–76)	27 (24–29)			38 (24–63)	No	8	
					taTME	44	66 (59–74)	26 (23– 28)			40 (31–48)			
Perdawood et al., 2016 (32)	Colorectal Disease	Denmark	2013-2015	Prospective	laTME	25	70 (4984)	26 (1938)	50 (2080)	8 (510)		Yes (propensity score matching)	8	
					taTME	25	70 (5476)	28 (1846)	50 (2070)	8 (410)				
Persiani et al., 2018 (33)	Dis Colon Rectum	Italy	2007-2017	Prospective	laTME	46	66.5 (28–86)	25.6 (18.8–33.4)	27 (3–80)			Yes (propensity score matching)	8	
					taTME	46	69 (36–94)	25 (19.1–32.8)	25 (8–75)					
Pontallier et al., 2016 (34)	Surg Endosc	France	2008-2012	Prospective	laTME	34	62 (35–82)	24.8 (18.3–38.3)	4 (1–8)		78	No	7	
					taTME	38	62 (39–81)	25.5 (17.3–33.2)	4 (1.5–8)		73			
Rasulov et al., 2016 (35)	Tech Coloproctol	Russia	2013-2015	Prospective	laTME	23	26.0(18.3–37.2)	60 (15–78)		7 Median (cm)	11.4	Yes (propensity score matching)	8	
					taTME	22	26.0(19.7–32.3)	56 (30–69)		6.5 Median (cm)	11.4			
Ren et al., 2021 (36)	Asian J Surg	China	2017-2019	Retrospective	laTME	32	67.16 ± 10.03	23.05 ± 2.70	4.14 ± 1.89			Yes (propensity score matching)	8	
					taTME	32	65.78 ± 12.37	22.87 ± 2.66	4.20 ± 1.20					
Roodbeen et al., 2019 (37)	Surg Endosc	Netherlands	2013-2017	Prospective	laTME	41	66.0±9.2	26.1± 4.0	43.0 (37.0–55.0)			Yes (propensity score matching)	7	
					taTME	41	62.5±10.7	26.7 ±1.9	46.5 (34.5–53.8)					
Rubinkiewicz et al., 2018 (38)	Cancer Manag Res	Poland	2012-2014	Prospective	laTME	35	60.3±10.2	27.1±4.71				Yes (propensity score matching)	8	
			2015-2018		taTME	35	64.3±10.1	26.1±4.09						
Rubinkiewicz et al., 2018 (39)	BMC Surg	Poland	2013-2017	Prospective	laTME	23	64 [58–67]	26.5 [23.8–30.6]				Yes (propensity score matching)	6	
					taTME	23	60 [51–67]	26 [22.8–29.7]						
Seow−En et al., 2018 (40)	Ann Acad Med Singap	Singapore	2012-2015	Prospective	RoTME	21		24 (22 – 26)	35 (21 – 48)		28 (22 – 38)	No	7	
					taTME	6		24 (20 – 27)	39 (23 – 61)		30 (29 – 35)			
Sun et al., 2022 (41)	Zhonghua Wei Chang Wai Ke Za Zhi	China	2014-2020	Retrospective	laTME	52	59±9	24.3±2.9			72	Yes (propensity score matching)	6	
					taTME	52	59±10	24.3±3.2			72			
Veltcamp Helbach et al., 2019 (42)	Surg Endosc	Netherlands	2010-2012	Retrospective	laTME	27	62.7 (59.6–65.7)	26.1 (25.1–27.3)			59.5(39.7–82.0)	Yes (propensity score matching)	7	
					taTME	27	68.0 (64.4–71.6)	27.6 (25.7–29.5)			20.0(6.6–44.4)			
Ye et al., 2021 (43)	Eur J Surg Oncol	China	2014-2019	Retrospective	laTME	70		22.7(±3.0)			20 (4–59)	Yes (propensity score matching)	8	
					taTME	70		23.5(±3.5)			18 (3–63)			
Zeng et al., 2020 (44)	Surgical Endoscopy and Other Interventional Techniques	China	2016-2018	Retrospective	laTME	133	56.1± 10.9	22.2±2.9	3.0± 1.3			No	8	
					taTME	128	56.1±11.2	22.5±3.1	3.2± 1.3					
Zeng et al., 2021 (45)	Dis Colon Rectum	China	2014-2018	Retrospective	laTME	171	59.1 ± 11.5	22.6 ± 3.4	3.0 ± 1.2		26 (15–36)	Yes (propensity score matching)	8	
					taTME	171	55.6 ± 12.6	22.9 ± 3.1	2.9 ± 1.2		26 (15–36)			
Zeng et al., 2022 (46)	Surg Endosc	China	2014-2017	Retrospective	laTME	208	58.3± 12.1	22.5±3.2	3.3± 1.2		15 (1–32)	No	7	
					taTME	104	57.2±11.9	22.6± 3.0	3.1± 1.2		17 (6–35)			
Zuhdy, 2020 (47)	J Laparoendosc Adv Surg Tech A	Egypt	2017-2019	Prospective	laTME	20	53.40 – 11.38	25.99 – 4.68					7	
					taTME	18	53.89 – 13.99	30.74 – 7.79						

Matching: 1 - Age; 2 - BMI; 3 - Tumor size; 4 - Tumor height; 5 - Follow-up. laTME, laparoscopic total mesorectal excision; RoTME, Robotic total mesorectal excision; taTME: transanal total mesorectal excision; NA, data not available. NOS score: Newcastle-Ottawa Scale score.

### 2.5 Statistical analysis

Statistical analysis was performed by Stata v.12.0 (Stata Corp LLC, College Station, TX, USA). For this meta-analysis, if the heterogeneity test was I^2^>50%, P<0.1, we used the random effect model; if the heterogeneity test was I^2^<50%, P>0.1, we used the fixed utility model. The combined r values and 95% confidence intervals (CIs) of each study were calculated, and the forest map displayed the characteristics of each study result. The quality of the included literature was evaluated using the Newcastle–Ottawa scale (NOS). Begg’s and Egger’s tests were used to test the publication bias. The P<0.05 was indicated as statistically significant.

## 3 Results

### 3.1 Eligible studies and study characteristics

**Figure 1:**
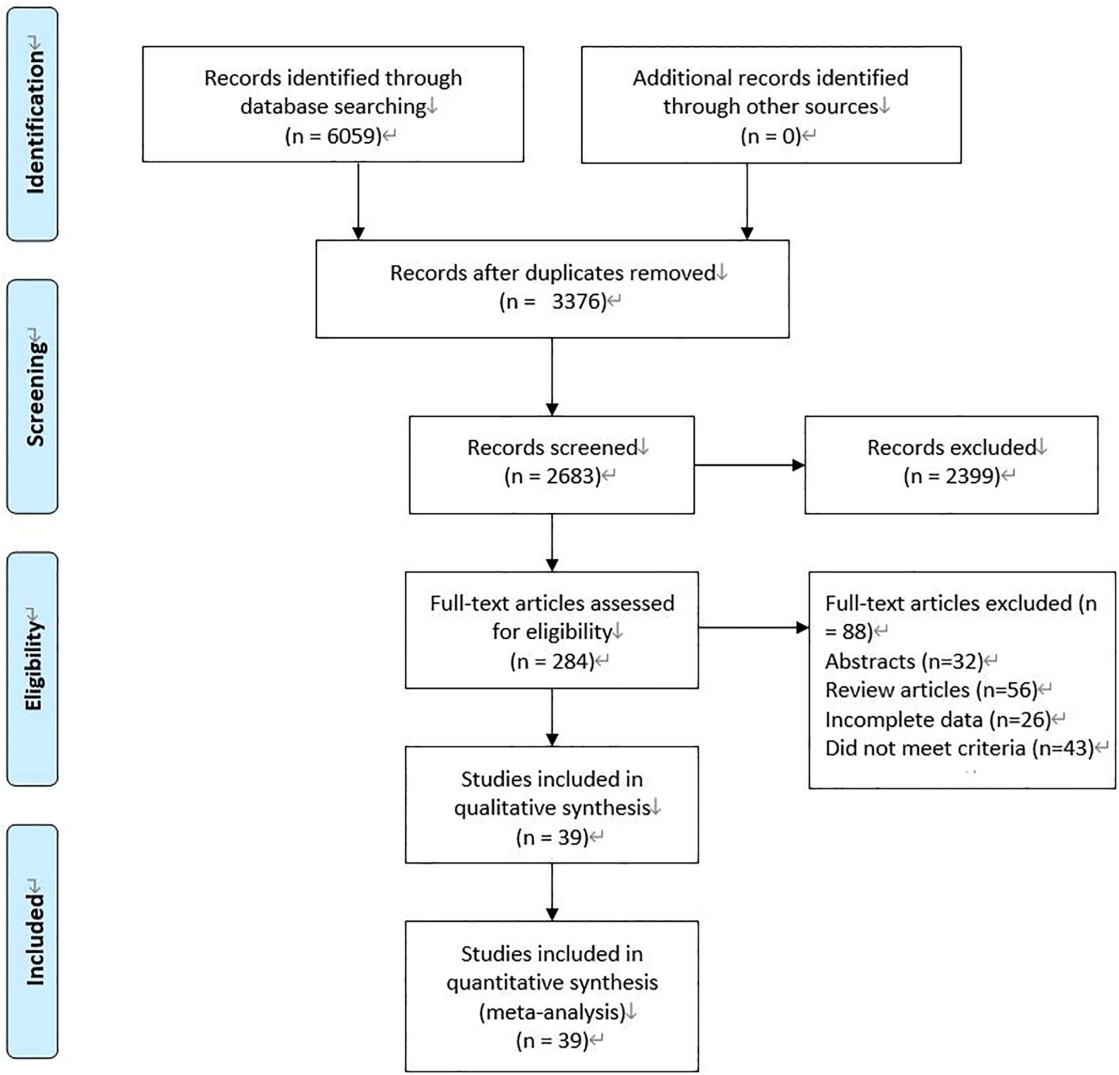
Flowchart for records selection process of the meta-analysis. (According to PRISMA template: Moher D, Liberati A, Tetzlaff J, Altman DG, The PRISMA Group (2009). Preferred Reporting Items for Systematic Reviews and Meta-Analyses: The PRISMA Statement. PLoS Med 6(7): e1000097. doi:10.1371/journal. Pmed 1000097).

We initially searched 6059 records. 3376 literature that was published repeatedly and cross-published were deleted. After reading the title and abstract, 2399 articles were excluded. After the remaining 284 pieces of literature were searched for full text, reading, and quality assessment, 39 pieces of literature (11010 patients: MiTME: 6268 vs TaTME: 4742) were eventually included (**Figure 1**). The detailed information on this literature was listed in **Table 1**.

### 3.2 Perioperative outcomes

**Figure 2:**
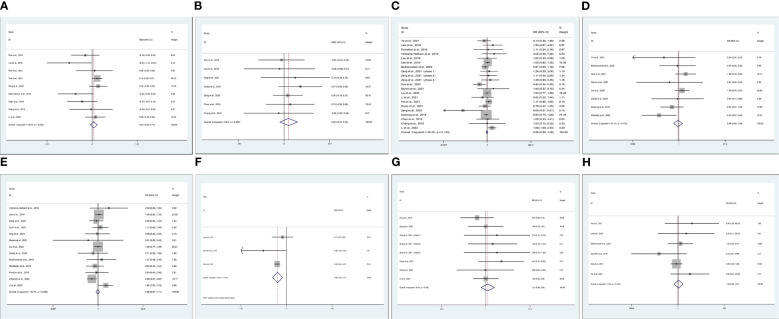
Meta-analysis of minimally invasive total mesorectal excision vs transanal total mesorectal excision for mid and low rectal cancer in **(A)** operation time, **(B)** estimated blood loss **(C)** postoperative hospital stays **(D)** over complications, **(E)** intraoperative or postoperative complications, **(F)** anastomotic leak rates, **(G)** anastomotic stenosis, **(H)** wound infection.

Data on operation time (OP) were reported in 21 studies (6, 14, 15, 17–21, 23, 25, 27, 30, 36, 38, 41, 44–49). Compared with TaTME, patients who underwent MiTME had no statistical difference (SMD -0.00; CI -0.06 to 0.06; I^2 = ^84.7%, P=0.885). Owing to high heterogeneity (I^2 = ^84.7%), we chose subgroup analysis. Compared with TaTME, patients who underwent RoTME or LaTME had no statistical difference (SMD -0.03; CI -0.37 to 0.31; I^2 = ^82.5%, P=0.866; SMD -0.18; CI -0.40 to 0.04; I^2 = ^86.0%, P=0.102). Sensitivity analysis and subgroup analysis cannot reduce heterogeneity. Therefore, we choose random effect model results (SMD -0.14; CI -0.31 to 0.33; I^2 = ^84.7%, P=0.116) (**Figure 2A**). We included 11 studies (6, 14, 15, 17, 19, 23, 25, 30, 36, 38, 44) about estimated blood loss (EBL). Compared with TaTME, patients who underwent MiTME had no statistical difference (SMD 0.00; CI -0.09 to 0.09; I^2 = ^61.2%, P=0.955). Owing to high heterogeneity (I^2 = ^61.2%**),** sensitivity analysis was carried out by Stata 12.0. After removing the studies by Grass et al (19) and Ong et al (30) as the sample that was “left out”, the pooled results did not change substantially but the heterogeneity was significantly reduced (SMD 0.05; CI -0.05 to 0.14; I^2 = ^48%, P=0.338) (**Figure 2B**). Data on postoperative hospital stays were reported in 7 studies (14, 15, 17, 19, 23, 30, 44). Compared with TaTME, patients who underwent MiTME had no statistical difference (SMD 0.08; CI -0.07 to 0.22; I^2 = ^0%, P=0.308) (**Figure 2C**).

Data on over complications were reported in 20 studies (14–16, 19–21, 23, 24, 26, 27, 29, 34, 41, 43–45, 49–51). Compared with TaTME, patients who underwent MiTME had no statistical difference (RR 0.98; CI 0.88 to 1.08; I^2 = ^25.4%, P=0.644) (**Figure 2D**). Compared with TaTME, patients who underwent MiTME had no statistical difference in intraoperative (RR 0.94; CI 0.69 to 1.29; I^2 = ^31.1%, P=0.712) (**Figure 2E-1**) or postoperative complications (RR 0.98; CI 0.87 to 1.11; I^2 = ^16.1%, P=0.789) (**Figure 2E-2**). Compared with TaTME, patients who underwent MiTME had less anastomotic leak rates (SMD -0.38; CI -0.59 to -0.17; I^2 = ^19.0%, P<0.0001) (**Figure 2F**), patients who underwent MiTME had no statistical difference in anastomotic stenosis (RR 0.85; CI 0.73 to 0.98; I^2 = ^7.4%, P=0.564) (**Figure 2G**), and patients who underwent MiTME had no statistical difference for wound infection (RR 1.08; CI 0.65 to 1.81; I^2 = ^1.9%, P=0.755) (**Figure 2H**).

### 3.3 Postoperative outcomes

**Figure 3:**
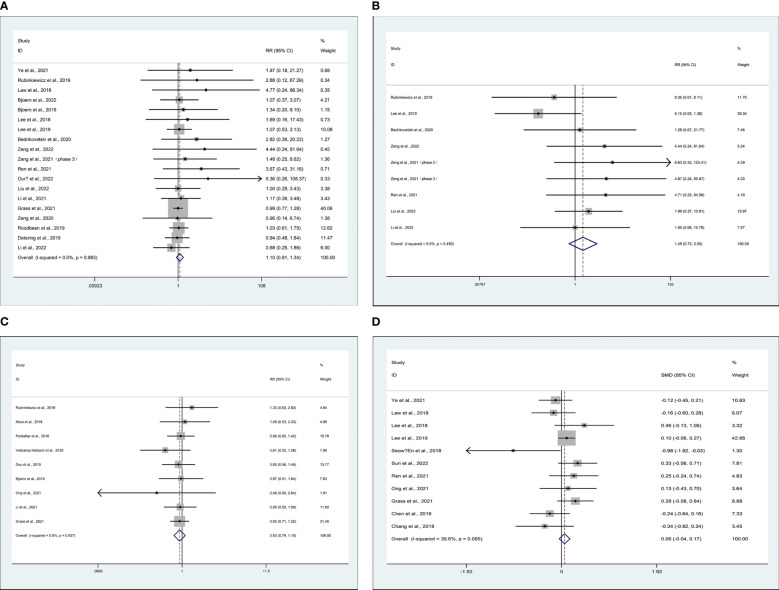
Meta-analysis of minimally invasive total mesorectal excision vs transanal total mesorectal excision for mid and low rectal cancer in **(A)** circumferential resection margin, **(B)** distal resection margin, **(C)** major low anterior resection syndrome, and **(D)** lymph node yield.

Data on circumferential resection margin (CRM) were reported in 19 studies (11–13, 16, 19, 23–27, 31, 36–38, 43, 44, 49). Compared with TaTME, patients who underwent MiTME had no statistical difference (RR 1.10; CI 0.91 to 1.34; I^2 = ^0%, P=0.322) (**Figure 3A**). Data on distal resection margin (DRM) were reported in 7 studies (24, 25, 27, 36, 38, 45, 46). Compared with TaTME, patients who underwent MiTME had no statistical difference (RR 1.49; CI 0.73 to 3.05; I^2 = ^0%, P=0.272) (**Figure 3B**). Data on major low anterior resection syndrome (LARS) were reported in 9 studies (12, 17, 19, 26, 28, 30, 34, 38, 50). Compared with TaTME, patients who underwent MiTME had no statistical difference (RR 0.93; CI 0.79 to 1.10; I^2 = ^0%, P=0.386) (**Figure 3C**). Data on lymph node yield were reported in 11 studies (14, 15, 19, 23, 24, 30, 36, 41, 43, 48, 49). Compared with TaTME, patients who underwent MiTME had no statistical difference (SMD 0.06; CI -0.04 to 0.17; I^2 = ^39.6%, P=0.249) (**Figure 3D**).

### 3.4 Oncological outcomes

**Figure 4:**
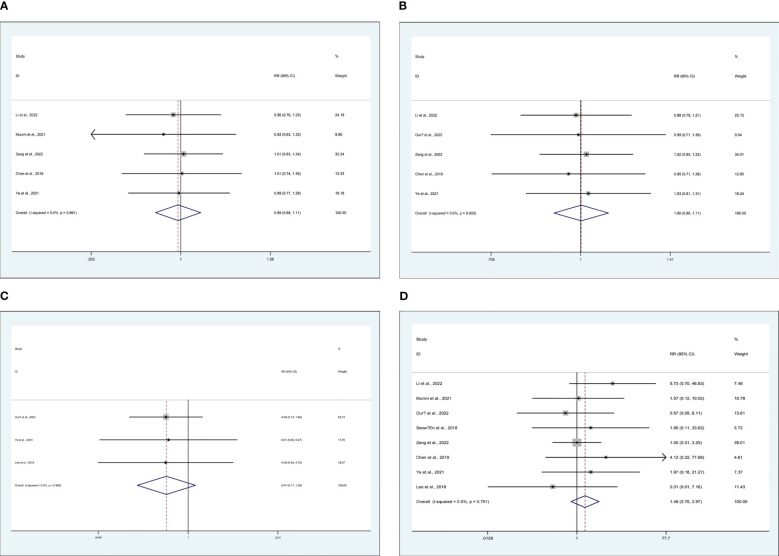
Meta-analysis of minimally invasive total mesorectal excision vs transanal total mesorectal excision for mid and low rectal cancer in **(A)** 2-year DFS rate, **(B)** 2-year OS rate, **(C)** distant metastasis rate, and **(D)** local recurrence rate.

5 studies recorded on 2-year disease-free survival (DFS) rate (15, 25, 29, 43, 46), 5 studies recorded on 2-year overall survival (OS) rate (15, 25, 31, 43, 46), 3 studies recorded on distant metastasis (23, 31, 43), and 8 studies recorded on local recurrence (15, 23, 25, 29, 31, 43, 46, 48). There are similarities between MiTME and TaTME for the 2-year DFS rate (RR 0.99; CI 0.88 to 1.11; I^2 = ^0%, P = 0.816) (**Figure 4A**), 2-year OS rate (RR 1.00; CI 0.90 to 1.11; I^2 = ^0%, P = 0.969) (**Figure 4B**), distant metastasis rate (RR 0.47; CI 0.17 to 1.29; I^2 = ^0%, P = 0.143) (**Figure 4C**), and local recurrence rate (RR 1.49; CI 0.75 to 2.97; I^2 = ^0%, P = 0.250) (**Figure 4D**).

## 4 Publication bias

We conducted publication bias on more than 15 included studies using Begg’s test. For OP, Begg’s test results revealed that t=-1.87, P=.075 in **Supplementary Figure 1A**. For over complications. Begg’s test results revealed that t=0.81, P=.427 in **Supplementary Figure 1B**. For the circumferential resection margin, Begg’s test results revealed that t=4.20, P=.001 in **Supplementary Figure 1C**. There is no publication bias except circumferential resection margin in the above.

## 5 Discussion

As TaTME has reported more and more in recent years, so has its controversy (52). The main focus is on whether TaTME can get better safety and efficacy with mid to low-rectal cancer in patients. The results of this study show that patients who underwent MiTME had fewer anastomotic leak rates. Compared with TaTME, patients who underwent MiTME had no statistical difference in OP, EBL, postoperative hospital stay, over complications, intraoperative complications, postoperative complications, anastomotic stenosis, wound infection, CRM, DRM, major LARS, lymph node yield, 2-year DFS rate, 2-year OS rate, distant metastasis rate, and local recurrence rate. The absence of heterogeneity in postoperative hospital stays, circular differential recovery margin, total recovery margin, major low adverse recovery syndrome, 2-year disease-free survival, 2-year overall survival rate, distance metastasis rate, and local recurrence rate indicates that these results are reliable. The slightly lower heterogeneity of postoperative hospital stays, over applications, intra-operational applications, postoperative applications, analytical leak rates, analytical stenosis, and weak node yield indicates that these results are relatively reliable. The heterogeneity of EBL is slightly higher, which may be related to different surgeons. The high heterogeneity of OP indicates the low reliability of these results.

CRM positive rate is a good evaluation index for tumor outcome (53). This study’s results suggest no significant difference in the positive rate of CRM, DRM, lymph node yield between TaTME and MITME. This indicates that there is no difference in the treatment effectiveness between the two. In secondary outcomes, there is no significant difference between the two in terms of OP, EBL, postoperative hospital stays, CRM, DRM, LARS, lymph node yield, and incidence of intraoperative and postoperative complications. However, it is expected to achieve better results with the technique becomes more proficient in the application of mid and low rectal cancer (54). For oncological outcomes, only a small portion of studies have reported differences in late local recurrence and survival between the two groups. The Zeng (46) et al.’s study was found that the local recurrence rate was 3.8% in both groups of patients and another study confirmed that local recurrence is only 3% after TaTME for rectal cancer (55). However, our research results showed that there was no difference in DFS, OS, distance metastasis rate, and local recurrence rate between the two groups at 2 years. Currently, larger RCT studies are underway (56), and more reliable results are expected.

Both types of rectal cancer surgery have a certain impact on a patient’s quality of life (57), mainly LARS (58). A study suggests that some patients develop severe LARS after TaTME (59). Another article found a low incidence of mild/severe LARS in patients after TaTME (60). There was no significant difference in LARS between the two groups in this study. It shows that the probability of anal sphincter injury function damage is not increased after the anal operation of TaTME. This conclusion also adds a strong backing for the application of TaTME.

Of course, our research also has some limitations: 1. The included studies are retrospective studies or prospective cohort studies, which will inevitably be affected by selection bias. 2. In terms of the baseline report of the cases included in the literature, only some of them were provided. Of course, we analyzed the baseline data that can be extracted from the included literature, but we still lacked the comprehensiveness of the data, and could not conduct subgroup analysis according to general characteristics, such as male-female ratio, BMI value, etc. 3. In the data analysis, although we conducted a sensitivity analysis on highly heterogeneous outcome indicators, some results did not identify the source of their heterogeneity. 4. In terms of analysis indicators, the long-term efficacy, such as local tumor recurrence rate, was not analyzed by subgroup according to the follow-up time, while only 5 articles were included in the 2-year DFS and 2-year OS, and the number of articles included in the analysis was insufficient. 5. At present, the follow-up time of various studies is limited, and not enough long-term efficacy data is provided for analysis. In terms of functional outcome data, only kinds of literature mention it and it is not uniformly quantified, which causes certain difficulties in analysis.

## 6 Conclusion

This study comprehensively and systematically evaluated the safety and efficacy of MiTME and TaTME in the treatment of mid to low rectal cancer through meta-analysis. There is no difference between the two except for patients with MiTME who have a lower anastomotic leakage rate, which provides some evidence-based reference for clinical practice. Of course, in the future, more scientific and rigorous conclusions need to be drawn from multi-center RCT research.

## Data availability statement

The datasets presented in this study can be found in online repositories. The names of the repository/repositories and accession number(s) can be found in the article/**Supplementary Material**.

## Author contributions

Conceptualization: LD, ZD. Data curation: LD, LY, ZC. Formal analysis: LD, LY. All authors contributed to the article and approved the submitted version.

## Supplementary material

Supplementary Figure 1 Egger's publication bias plot to detect publication bias.

## References

[1] Sung H Ferlay J Siegel RL Laversanne M Soerjomataram I Jemal A . Global cancer statistics 2020: GLOBOCAN estimates of incidence and mortality worldwide for 36 cancers in 185 countries. CA Cancer J Clin (2021) 71:209–49. doi: 10.3322/caac.21660 33538338

[2] Nagtegaal ID van de Velde CJ van der Worp E Kapiteijn E Quirke. P van Krieken. JH . Macroscopic evaluation of rectal cancer resection specimen: clinical significance of the pathologist in quality control. J Clin Oncol (2002) 20:1729–34. doi: 10.1200/JCO.2002.07.010 11919228

[3] . An international multicentre prospective audit of elective rectal cancer surgery; operative approach versus outcome, including transanal total mesorectal excision (TaTME). Colorectal Dis (2018) 20(Suppl 6):33–46. doi: 10.1111/codi.14376 30255642

[4] Targarona EM Balague C Pernas JC Martinez C Berindoague R Gich I . Can we predict immediate outcome after laparoscopic rectal surgery? multivariate analysis of clinical, anatomic, and pathologic features after 3-dimensional reconstruction of the pelvic anatomy. Ann Surg (2008) 247:642–9. doi: 10.1097/SLA.0b013e3181612c6a 18362627

[5] Di Saverio S Gallo G Davies RJ Bergamaschi R Wheeler J Sileri P . Robotic-assisted transanal total mesorectal excision for rectal cancer: more questions than answers. Tech Coloproctol (2021) 25:987–8. doi: 10.1007/s10151-020-02402-7 33449254

[6] Ose. I Perdawood. SK . A nationwide comparison of short-term outcomes after transanal, open, laparoscopic, and robot-assisted total mesorectal excision. Colorectal Dis (2021) 23:2671–80. doi: 10.1111/codi.15809 34273239

[7] Young. M Pigazzi. A . Total mesorectal excision: open, laparoscopic or robotic. Recent Results Cancer Res (2014) 203:47–55. doi: 10.1007/978-3-319-08060-4_6 25102999

[8] Creavin B Kelly ME Ryan É J Ryan. OK Winter. DC . Oncological outcomes of laparoscopic versus open rectal cancer resections: meta-analysis of randomized clinical trials. Br J Surg (2021) 108:469–76. doi: 10.1093/bjs/znaa154 33748848

[9] Page MJ McKenzie JE Bossuyt PM Boutron I Hoffmann TC Mulrow CD . The PRISMA 2020 statement: an updated guideline for reporting systematic reviews. Bmj (2021) 372:n71. doi: 10.1136/bmj.n71 33782057 PMC8005924

[10] Alhanafy MK Park SS Park SC Park B Kim MJ Sohn DK . Early experience with transanal total mesorectal excision compared with laparoscopic total mesorectal excision for rectal cancer: a propensity score-matched analysis[J]. Dis Colon Rectum (2020) 63:1500–10. 10.1097/DCR.0000000000001725 33044291

[11] Bedrikovetski S Dudi-Venkata NN Kroon HM Moore JW Hunter. RA Sammour. T . Outcomes of minimally invasive versus open proctectomy for rectal cancer: a propensity-matched analysis of bi-national colorectal cancer audit data. Dis Colon Rectum (2020) 63:778–87. doi: 10.1097/DCR.0000000000001654 32109916

[12] Bjoern MX Nielsen. S Perdawood. SK . Quality of life after surgery for rectal cancer: a comparison of functional outcomes after transanal and laparoscopic approaches. J Gastrointest Surg (2019) 23:1623–30. doi: 10.1007/s11605-018-4057-6 30603861

[13] Bjoern MX Clausen FB Seiersen M Bulut O Bech-Knudsen F Jansen JE . Quality of life and functional outcomes after transanal total mesorectal excision for rectal cancer-results from the implementation period in Denmark. Int J Colorectal Dis (2022) 45:2197–2202. doi: 10.1007/s00384-022-04219-2 35960389

[14] Chang. TC Kiu. KT . Transanal total mesorectal excision in lower rectal cancer: comparison of short-term outcomes with conventional laparoscopic total mesorectal excision. J Laparoendosc Adv Surg Tech A (2018) 28:365–9. doi: 10.1089/lap.2017.0520 29190178

[15] Chen Y-T Kiu K-T Yen. M-H Chang. T-C . Comparison of the short-term outcomes in lower rectal cancer using three different surgical techniques: transanal total mesorectal excision (TME), laparoscopic TME, and open TME. Asian J Surg (2019) 42:674–80. doi: 10.1016/j.asjsur.2018.09.008 30318319

[16] Detering R Roodbeen SX van Oostendorp SE Dekker J-WT Sietses C Bemelman WA . Three-year nationwide experience with transanal total mesorectal excision for rectal cancer in the Netherlands: a propensity score-matched comparison with conventional laparoscopic total mesorectal excision. J Am Coll Surgeons (2019) 228:235–244.e1. doi: 10.1016/j.jamcollsurg.2018.12.016 30639298

[17] Dou R Sun W Luo S Hou Y Zhang. C Kang. L . Comparison of postoperative bowel function between patients undergoing transanal and laparoscopic total mesorectal excision. Zhonghua Wei Chang Wai Ke Za Zhi (2019) 22:246–54. doi: 10.3760/cma.j.issn.16710274.2019.03.011 30919377

[18] Fernandez-Hevia M Delgado S Castells A Tasende M Momblan D del Gobbo GD . Transanal total mesorectal excision in rectal cancer short-term outcomes in comparison with laparoscopic surgery. Ann Surg (2015) 261:221–7. doi: 10.1097/SLA.0000000000000865 25185463

[19] Grass J-K Persiani R Tirelli F Chen C-C Caricato M Pecorino A . Robotic versus transanal total mesorectal excision in sexual, anorectal, and urinary function: a multicenter, prospective, observational study. Int J colorectal Dis (2021) 36:2749–61. doi: 10.1007/s00384-021-04030-5 PMC8589758 34537862

[20] Hol JC Burghgraef TA Rutgers MLW Crolla RMPH van Geloven NAW Hompes R . Comparison of laparoscopic versus robot-assisted versus transanal total mesorectal excision surgery for rectal cancer: a retrospective propensity score-matched cohort study of short-term outcomes. Br J Surg (2021) 108:1380–7. doi: 10.1093/bjs/znab233 34370834

[21] Jang HB Kang SB Lee H Choi. BJ Lee. SC . Anastomotic leakage and chronic presacral sinus after transanal total mesorectal excision (taTME) for rectal cancer: a comparative study to laparoscopic TME. Asian J Surg (2021) 277:1–6. doi: 10.1016/j.asjsur.2021.11.009 34801358

[22] Law W Foo DCC . Comparison of early experience of robotic and transanal total mesorectal excision using propensity score matching[J]. Surg Endoscopy (2019) 33:757–63. 10.1007/s00464-018-6340-8 30014329

[23] Lee KY Shin JK Park YA Yun SH Huh JW Cho YB . Transanal endoscopic and transabdominal robotic total mesorectal excision for mid-to-Low rectal cancer: comparison of short-term postoperative and oncologic outcomes by using a case-matched analysis. Ann Coloproctol (2018) 34:29–35. doi: 10.3393/ac.2018.34.1.29 29535985 PMC5847400

[24] Lee L de Lacy B Gomez Ruiz M Liberman AS Albert MR Monson JRT . A multicenter matched comparison of transanal and robotic total mesorectal excision for mid and low-rectal adenocarcinoma. Ann Surg (2019) 270:1110–6. doi: 10.1097/SLA.0000000000002862 29916871

[25] Li Z Xiao J Hou Y Zhang X Jie H Liu H . Transanal versus laparoscopic total mesorectal excision in Male patients with low tumor location after neoadjuvant therapy: a propensity score-matched cohort study. Gastroenterol Res Pract (2022) 2022:2387464. doi: 10.1155/2022/2387464 35265121 PMC8898864

[26] Li Y Bai X Niu B Zhou J Qiu H Xiao Y . A prospective study of health related quality of life, bowel and sexual function after TaTME and conventional laparoscopic TME for mid and low rectal cancer. Tech Coloproctol (2021) 25:449–59. doi: 10.1007/s10151-020-02397-1 33646454

[27] Liu H Zeng Z Zhang H Wu M Ma D Wang Q . Morbidity, mortality, and pathologic outcomes of transanal versus laparoscopic total mesorectal excision for rectal cancer short-term outcomes from a multicenter randomized controlled trial. Ann Surg (2022) 37:1997–2011. doi: 10.1097/SLA.0000000000005523 PMC9762710 35815886

[28] Mora L Zarate A Serra-Aracil X Pallisera A Serra.S. Navarro-Soto. [Functional impairment S . And quality of life after rectal cancer surgery]. Cir Cir (2018) 86:140–7. doi: 10.24875/CIRU.M18000022 29809186

[29] Munini M Popeskou SG Galetti K Roesel R Mongelli. F Christoforidis. D . Transanal (TaTME) vs. laparoscopic total mesorectal excision for mid and low rectal cancer: a propensity score-matched analysis of early and long-term outcomes. Int J Colorectal Dis (2021) 36:2271–9. doi: 10.1007/s00384-021-04019-0 34467413

[30] Ong GK Tsai B Patron RL Johansen O Lane F Melbert RB . Transanal total mesorectal excision achieves equivalent oncologic resection compared to laparoscopic approach, but with functional consequences. Am J Surg (2021) 221:566–9. doi: 10.1016/j.amjsurg.2020.11.013 33208226

[31] Ourô S Ferreira M Roquete. P Maio. R . Transanal versus laparoscopic total mesorectal excision: a comparative study of long-term oncological outcomes. Tech Coloproctol (2022) 26:279–90. doi: 10.1007/s10151-022-02570-8 35050434

[32] Perdawood SK Khefagie GAA . Transanal vs laparoscopic total mesorectal excision for rectal cancer: initial experience from Denmark[J]. Colorectal Dis Off J Assoc Coloproctology Great Britain Ireland (2016) 18:51–8. 10.1111/codi.13225 26603786

[33] Persiani R Biondi A Pennestrì F Fico V De Simone V Tirelli F . Transanal total mesorectal excision vs laparoscopic total mesorectal excision in the treatment of low and middle rectal cancer: a propensity score matching analysis[J]. Dis Colon Rectum (2018) 61:809–16. 10.1097/DCR.0000000000001063 29771810

[34] Pontallier A Denost Q Van Geluwe B Adam JP Celerier. B Rullier. E . Potential sexual function improvement by using transanal mesorectal approach for laparoscopic low rectal cancer excision. Surg Endosc (2016) 30:4924–33. doi: 10.1007/s00464-016-4833-x 26944728

[35] Rasulov AO Mamedli ZZ Gordeyev SS Kozlov NA Dzhumabaev HE . Short-term outcomes after transanal and laparoscopic total mesorectal excision for rectal cancer[J]. Tech Coloproctol (2016) 20:227–34. 10.1007/s10151-015-1421-3 26794213

[36] Ren J Liu S Luo H Wang. B Wu. F . Comparison of short-term efficacy of transanal total mesorectal excision and laparoscopic total mesorectal excision in low rectal cancer. Asian J Surg (2021) 44:181–5. doi: 10.1016/j.asjsur.2020.05.007 32461015

[37] Roodbeen SX Penna M Mackenzie H Kusters M Slater A Jones OM . Transanal total mesorectal excision (TaTME) versus laparoscopic TME for MRI-defined low rectal cancer: a propensity score-matched analysis of oncological outcomes. Surg Endosc (2019) 33:2459–67. doi: 10.1007/s00464-018-6530-4 PMC6647375 30350103

[38] Rubinkiewicz M Nowakowski M Wierdak M Mizera M Dembiński M Pisarska M . Transanal total mesorectal excision for low rectal cancer: a case-matched study comparing TaTME versus standard laparoscopic TME. Cancer Manag Res (2018) 10:5239–45. doi: 10.2147/CMAR.S181214 PMC6219401 30464621

[39] Rubinkiewicz M Nowakowski M Wierdak M Mizera M Dembiński M Pisarska M . Transanal total mesorectal excision for low rectal cancer: a case-matched study comparing TaTME versus standard laparoscopic TME[J]. Cancer Manag Res (2018) 10:5239–45. 10.2147/CMAR.S181214 PMC6219401 30464621

[40] Seow-En I Seow-Choen F . An initial experience comparing robotic total mesorectal excision (RTME) and transanal total mesorectal excision (taTME) for low rectal Tumours[J]. Ann Acad Med Singap (2018) 47:188–90. 29911735

[41] Sun R Cong L Qiu HZ Lin GL Wu B Niu BZ . [Safety and prognosis analysis of transanal total mesorectal excision versus laparoscopic mesorectal excision for mid-low rectal cancer]. Zhonghua Wei Chang Wai Ke Za Zhi (2022) 25:522–30. doi: 10.3760/cma.j.cn4415302021081100321 35754217

[42] Veltcamp Helbach JM Koedam TWA Knol JJ Velthuis S Bonjer HJ Tuynman JB . Quality of life after rectal cancer surgery: differences between laparoscopic and transanal total mesorectal excision[J]. Surg Endosc (2019) 33:79–87. 29967994 10.1007/s00464-018-6276-z PMC6336756

[43] Ye J Tian Y Li F van Oostendorp S Chai Y Tuynman J . Comparison of transanal total mesorectal excision (TaTME) versus laparoscopic TME for rectal cancer: a case matched study. Eur J Surg Oncol (2021) 47:1019–25. doi: 10.1016/j.ejso.2020.11.131 33309105

[44] Zeng ZW Luo SL Chen JJ Cai YH Zhang. XW Kang. L . Comparison of pathological outcomes after transanal versus laparoscopic total mesorectal excision: a prospective study using data from randomized control trial. Surg Endoscopy Other Interventional Techniques (2020) 34:3956–62. doi: 10.1007/s00464-019-07167-1 31586244

[45] Zeng Z Liu Z Huang L Liu H Jie H Luo S . Transanal total mesorectal excision in mid-low rectal cancer: evaluation of the learning curve and comparison of short-term results with standard laparoscopic total mesorectal excision. Dis Colon Rectum (2021) 64:380–8. doi: 10.1097/DCR.0000000000001816 33394779

[46] Zeng Z Liu Z Luo S Liang Z Huang L Ruan L . Three-year outcomes of transanal total mesorectal excision versus standard laparoscopic total mesorectal excision for mid and low rectal cancer. Surg Endosc (2022) 36:3902–10. doi: 10.1007/s00464-021-08707-4 34448933

[47] Zuhdy M Elmore U Shams N Hegazy MAF Roshdy S Eldamshety O . Transanal versus laparoscopic total mesorectal excision: a comparative prospective clinical trial from two centers. J Laparoendosc Adv Surg Tech A (2020) 30:769–76. doi: 10.1089/lap.2019.0828 32240035

[48] Seow-En. I Seow-Choen. F . An initial experience comparing robotic total mesorectal excision (RTME) and transanal total mesorectal excision (taTME) for low rectal tumours. Ann Acad Med Singap (2018) 47:188–90. doi: 10.47102/annals-acadmedsg.V47N5p188 29911735

[49] Law. WL Foo. DCC . Comparison of early experience of robotic and transanal total mesorectal excision using propensity score matching. Surg Endosc (2019) 33:757–63. doi: 10.1007/s00464-018-6340-8 30014329

[50] Veltcamp Helbach M Koedam TWA Knol JJ Diederik A Spaargaren GJ Bonjer HJ . Residual mesorectum on postoperative magnetic resonance imaging following transanal total mesorectal excision (TaTME) and laparoscopic total mesorectal excision (LapTME) in rectal cancer. Surg Endosc (2019) 33:94–102. doi: 10.1007/s00464-018-6279-9 29967990 PMC6336750

[51] Bednarski BK . Minimally invasive rectal surgery: laparoscopy, robotics, and transanal approaches. J Surg Oncol (2020) 122:78–84. doi: 10.1002/jso.25925 32291771

[52] Jiang TY Ma. JJ Zheng. MH . Controversies and consensus in transanal total mesorectal excision (taTME): is it a valid choice for rectal cancer? J Surg Oncol (2021) 123(Suppl 1):S59–s64. doi: 10.1002/jso.26340 33650698

[53] Tilney HS Rasheed S Northover. JM Tekkis. PP . The influence of circumferential resection margins on long-term outcomes following rectal cancer surgery. Dis Colon Rectum (2009) 52:1723–9. doi: 10.1007/DCR.0b013e3181b54fbd 19966604

[54] Francis N Penna M Mackenzie H Carter. F Hompes. R . Consensus on structured training curriculum for transanal total mesorectal excision (TaTME). Surg Endosc (2017) 31:2711–9. doi: 10.1007/s00464-017-5562-5 28462478

[55] Roodbeen SX Spinelli A Bemelman WA Di Candido F Cardepont M Denost Q . Local recurrence after transanal total mesorectal excision for rectal cancer: a multicenter cohort study. Ann Surg (2021) 274:359–66. doi: 10.1097/SLA.0000000000003757 31972648

[56] Deijen CL Velthuis S Tsai A Mavroveli S de Lange-de Klerk ES Sietses C . COLOR III: a multicentre randomised clinical trial comparing transanal TME versus laparoscopic TME for mid and low rectal cancer. Surg Endosc (2016) 30:3210–5. doi: 10.1007/s00464-015-4615-x PMC4956704 26537907

[57] Frick MA Vachani CC Hampshire MK Bach C Arnold-Korzeniowski K Metz JM . Survivorship after lower gastrointestinal cancer: patient-reported outcomes and planning for care. Cancer (2017) 123:1860–8. doi: 10.1002/cncr.30527 28055110

[58] Koedam TW van Ramshorst GH Deijen CL Elfrink AK Meijerink WJ Bonjer HJ . Transanal total mesorectal excision (TaTME) for rectal cancer: effects on patient-reported quality of life and functional outcome. Tech Coloproctol (2017) 21:25–33. doi: 10.1007/s10151-016-1570-z 28044239 PMC5285410

[59] Tirelli F Lorenzon L Biondi A Neri I Santoro. G Persiani. R . Functional outcomes after transanal total mesorectal excision (TaTME): a random forest analysis to predict patients' outcomes. Tech Coloproctol (2023), 1–10. doi: 10.1007/s10151-023-02775-5 PMC9985820 36871281

[60] De Simone V Persiani R Biondi A Litta F Parello A Campennì P . One-year evaluation of anorectal functionality and quality of life in patients affected by mid-to-low rectal cancer treated with transanal total mesorectal excision. Updates Surg (2021) 73:157–64. doi: 10.1007/s13304-020-00919-y 33161532

