# Supplementary figures and images for: A systematic review and meta-analysis of minimally invasive total mesorectal excision versus transanal total mesorectal excision for mid and low rectal cancer
Source: Front Oncol. 2023 Jun 12;13:1167200. doi: 10.3389/fonc.2023.1167200 (PMC10291686; doi:10.3389/fonc.2023.1167200)

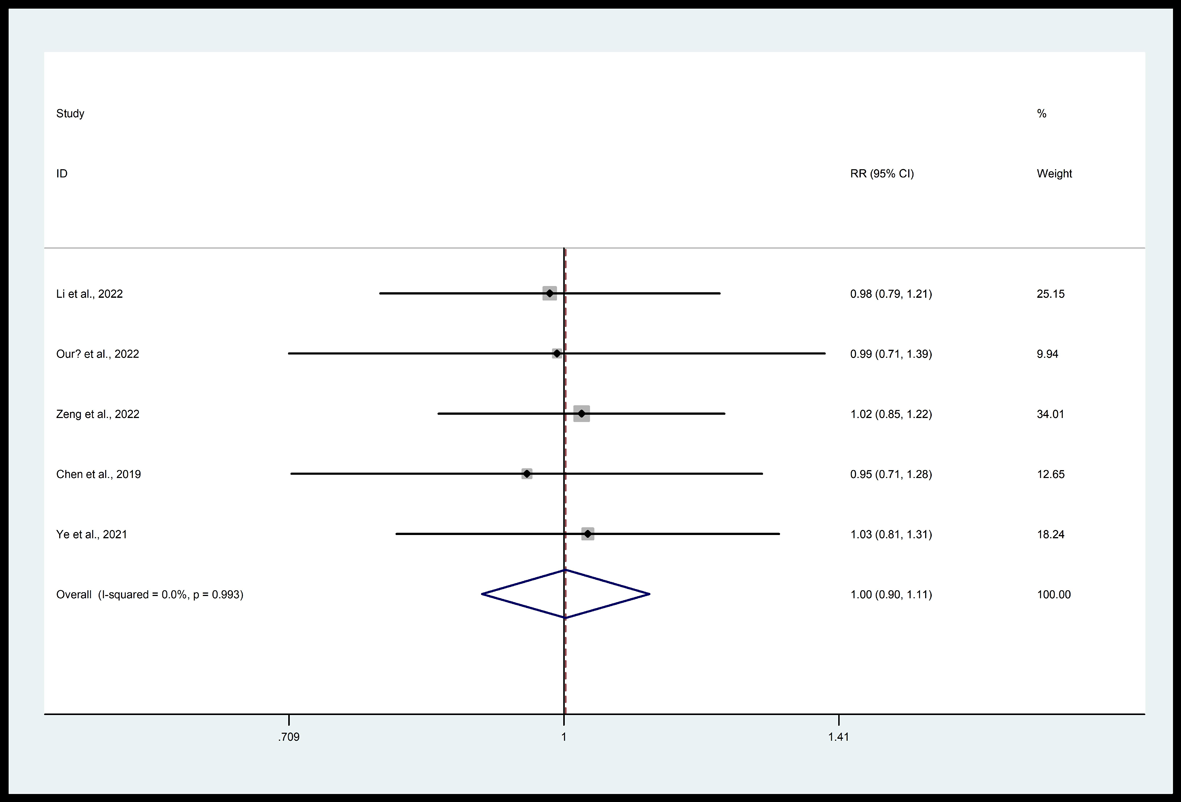

Supplement: Supplementary Figure 1 — Egger's publication bias plot to detect publication bias. [file Image_1.tif]

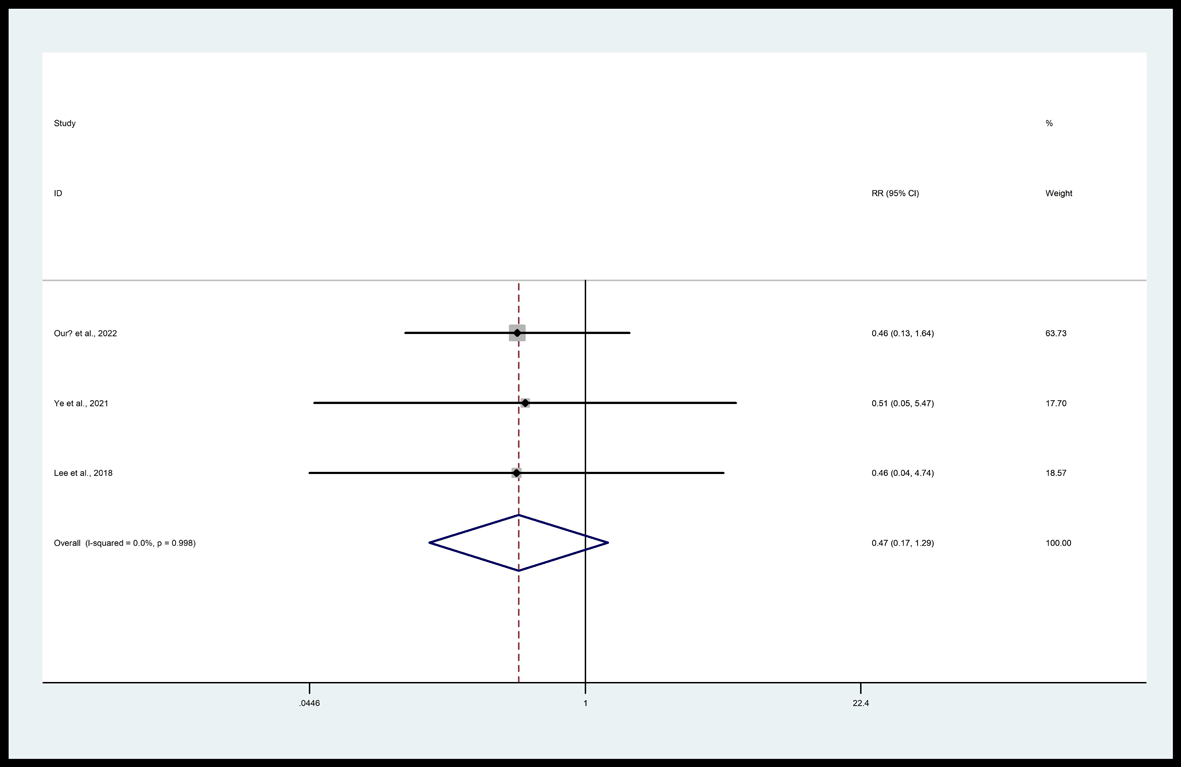

Supplement: Supplementary file 2 [file Image_2.tif]

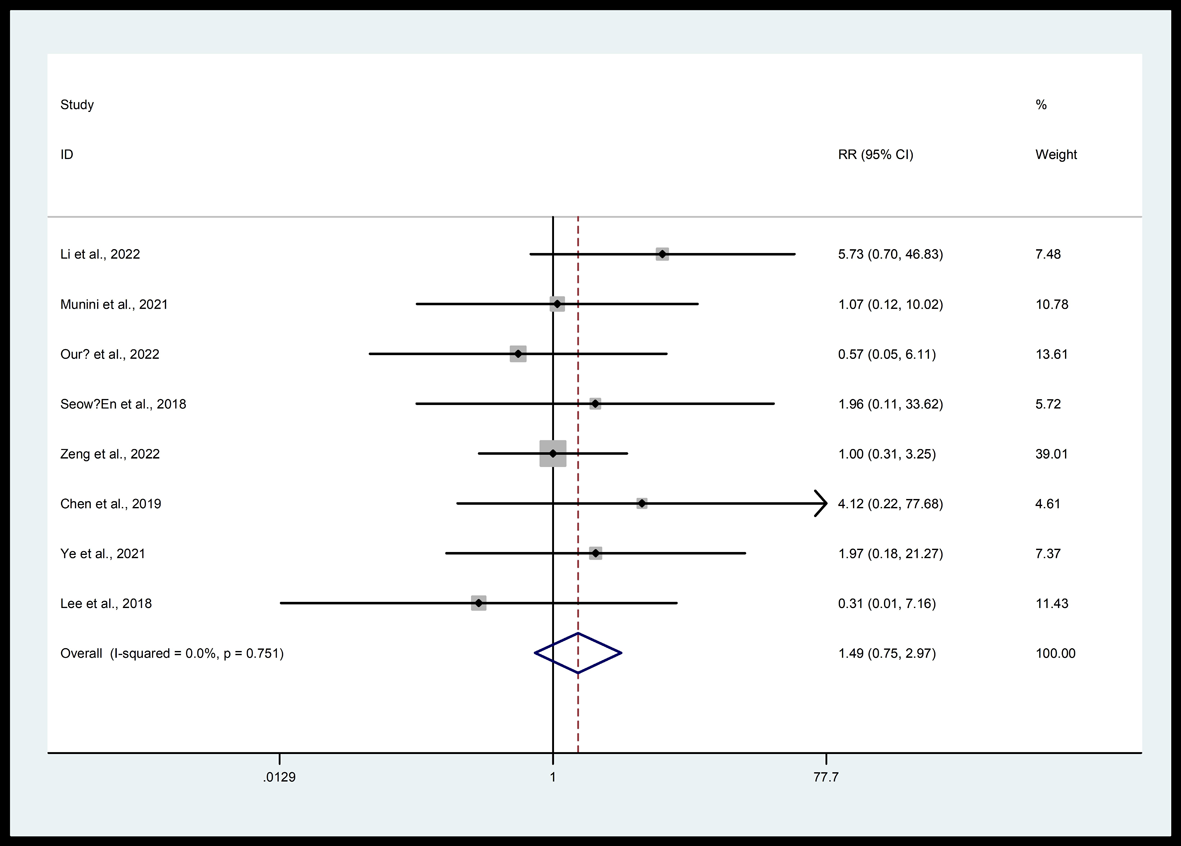

Supplement: Supplementary file 3 [file Image_3.tif]
